# Prognosis prediction by urinary liver-type fatty acid-binding protein in patients in the intensive care unit admitted from the emergency department: A single-center, historical cohort study

**DOI:** 10.62838/jccm-2026-0008

**Published:** 2026-04-30

**Authors:** Hirozumi Okuda, Hideki Asai, Koji Yamamoto, Keita Miyazaki, Hidetada Fukushima, Keigo Saeki

**Affiliations:** Department of Emergency and Critical Care Medicine, Nara Medical University, Kashihara City, Japan; Department of Epidemiology, Nara Medical University, Kashihara City, Japan

**Keywords:** emergency department admission, intensive care unit, liver-type fatty acid-binding protein, prognosis prediction

## Abstract

**Introduction:**

Early risk stratification of critically ill patients is essential for optimizing intensive care unit (ICU) resource allocation and treatment decisions. Urinary liver-type fatty acid-binding protein (L-FABP) is a simple, noninvasive biomarker that may provide real-time information on organ dysfunction. However, its prognostic utility in patients admitted to the ICU from the emergency department remains unclear.

**Aim of the study:**

The aim of this study was to evaluate the prognostic value of L-FABP levels measured shortly after ICU admission in predicting 28-day mortality among patients admitted from the emergency department.

**Methods:**

This single-center retrospective observational study included patients admitted to the ICU between December 2020 and August 2022. Urinary L-FABP concentrations were measured at ICU admission (T0) and 3 hours later (T3). The primary outcome was 28-day in-hospital mortality. Prognostic performance was assessed using receiver operating characteristic curves and Cox proportional hazards models with inverse probability of treatment weighting. Results were compared with Acute Physiology and Chronic Health Evaluation (APACHE) II, Sequential Organ Failure Assessment (SOFA) scores, and lactate levels.

**Results:**

Data of 118 patients were included in the final analysis. Urinary L-FABP levels at T3 showed the highest AUC for predicting 28-day mortality (area under the curve [AUC] = 0.873), compared with APACHE II (AUC = 0.801), SOFA (AUC = 0.753), and the lactate level (AUC = 0.734). An elevated L-FABP (T3) level was independently associated with increased mortality (hazard ratio [HR] = 8.60, 95% confidence interval [CI]: 1.02–72.64, P = 0.047). The T3/T0 ratio showed only modest predictive value (AUC = 0.623).

**Conclusions:**

Urinary L-FABP levels measured 3 hours after ICU admission were an independent predictor of short-term mortality. The marker’s simplicity and bedside applicability suggest its potential utility not only in ICUs but also in emergency departments and triage decision-making.

## Introduction

Intensive care units (ICUs) provide critical care for patients with life-threatening conditions, and early risk stratification is essential in guiding treatment and optimizing resource allocation. Conventional scoring systems, such as the Acute Physiology and Chronic Health Evaluation II (APACHE II) and Sequential Organ Failure Assessment (SOFA), are widely used for this purpose [1,2,3,4,5]. Although these tools are valuable when appropriately applied, the early phase of ICU admission is often marked by urgency and limited time, making it challenging for clinicians to solely rely on complex and time-consuming scoring systems.

In many countries, including Japan, the number of board-certified intensivists is limited, and ICU care is frequently provided in open ICU models [6,7]. In these settings, critically ill patients are managed by various non-intensivist physicians, such as emergency physicians, internists, surgeons, and anesthesiologists, who may have limited time for continuous patient assessment. Therefore, a simple, rapid, and objective prognostic biomarker is highly desirable, especially during the early phase of ICU admission, when clinical decisions must be made quickly under high-pressure conditions.

Liver-type fatty acid-binding protein (L-FABP) is a urinary biomarker for detecting acute kidney injury and intestinal ischemia. It is non-invasively measurable and has a short half-life, and it reflects systemic inflammation and organ dysfunction in near real-time. Although emerging evidence supports its prognostic value in critically ill patients, little is known about its utility in patients admitted to the ICU from the emergency department. Furthermore, most prior studies have focused on medical ICU patients with chronic conditions. In contrast, our study uniquely evaluated emergency ICU admissions primarily because of acute external causes such as trauma and poisoning, a population that has been underrepresented in L-FABP research.

This study aimed to evaluate the prognostic value of urinary L-FABP concentrations measured at ICU admission and 3 hours post-admission and to compare its performance with that of conventional prognostic indicators such as APACHE II, SOFA scores, and lactate levels.

## Materials and methods

### Study design and the primary outcome

This single-center, historical cohort study was conducted at the Advanced Emergency and Critical Care Center of our hospital from December 1, 2020, to August 31, 2022. The primary outcome was all-cause mortality within 28 days of ICU admission. The study protocol was approved by the Ethics Committee of our hospital (Approval No. 1798), and the study was conducted in accordance with the principles of the Declaration of Helsinki. Patients who met the inclusion criteria provided written informed consent.

### Inclusion and exclusion criteria

All patients admitted to the ICU of our hospital during the study period were screened for eligibility. To avoid collecting specimens solely for research purposes, only patients with a urinary catheter and an indwelling arterial line, both of which were placed as part of standard clinical management, were included. The exclusion criteria were: (1) age < 18 years, (2) known anuria before ICU admission due to chronic kidney disease or maintenance dialysis, and (3) confirmed or suspected coronavirus disease (COVID-19, excluded for biosafety reasons related to specimen collection, storage, or handling).

### Measurement of urinary L-FABP and other variables

Urine samples were collected at ICU admission (T0) and 3 hours later (T3). Each sample was placed in a sterile plastic container immediately after collection and stored at −80°C. Urinary L-FABP concentrations were measured using an enzyme-linked immunosorbent assay kit (L-FABP; CMIC Holdings Co., Ltd., Tokyo, Japan) in batch analyses outsourced to an external laboratory. Hourly urine output was monitored, and all samples used for analysis were confirmed to contain urine excreted within 1 hour of collection. Bedside nurses performed sample collection.

Clinical data were extracted from electronic medical records and included age, sex, height, weight, comorbidities, laboratory test results, arterial blood gas findings, APACHE II score, SOFA scores, and primary diagnosis.

### Study outcomes

The primary outcome was all-cause mortality within 28 days of ICU admission and its association with urinary L-FABP concentrations measured at T0 and T3.

Receiver operating characteristic (ROC) curves were generated for L-FABP levels at both time points, and the area under the curve (AUC) was calculated. These values were compared with conventional prognostic indicators, including the APACHE II, SOFA score, and arterial lactate concentration. Optimal cutoff values for each marker were determined using the Youden Index, and the potential prognostic utility of these markers was assessed.

The secondary outcome was the prognostic value of changes in urinary L-FABP levels over time, evaluated using the ratio (T3/T0) of the L-FABP concentration between the two measurements.

### Statistical analysis

Continuous variables were expressed as medians with interquartile ranges (IQRs), and categorical variables were expressed as counts and percentages. Group comparisons for continuous variables were performed using the Mann–Whitney U test. The prognostic performance of each marker was evaluated using ROC curve analysis, and AUC values were calculated for each predictor. The Youden index was used to determine optimal cut-off values. The prognostic performance of each marker was evaluated using ROC curve analysis, and AUC values were calculated for each predictor. The Youden index was used to determine optimal cut-off values. Pairwise comparisons of AUCs were performed using DeLong’s test for correlated ROC curves, with adjustments for multiple comparisons using the Bonferroni correction and the false discovery rate method. Given the exploratory nature of this analysis, differences in AUCs were primarily interpreted descriptively.

Kaplan–Meier survival curves were generated to compare 28-day survival between groups stratified by the cutoff value, and statistical significance was assessed using the log-rank test. To adjust for potential confounders, a Cox proportional hazards model was constructed using inverse probability of treatment weighting (IPTW) based on propensity scores for age, gender, weight, albumin level, ICU admission time, lactate level, APACHE II scores, and SOFA scores. Hazard ratios (HRs) and 95% confidence intervals (CIs) were calculated to estimate the association between L-FABP levels and 28-day mortality. Covariate balance after IPTW was assessed using standardized mean differences (SMDs), with an absolute SMD of <0.1 considered indicative of adequate balance.

All statistical analyses were performed using R software (version 4.3.3). A two-tailed p-value of <0.05 was considered statistically significant. Although a formal sample size calculation was not performed owing to the retrospective design, we conducted a post-hoc power analysis. Based on the observed effect size (HR = 8.60), sample size (n = 118), and α = 0.05, the estimated power was approximately 82%.

## Results

From December 1, 2020, to August 31, 2022, 774 patients were admitted to the ICU of the Advanced Emergency and Critical Care Center at our hospital. Among them, 138 patients who met the inclusion criteria and provided consent were enrolled after excluding those with confirmed or suspected COVID-19. However, 20 cases were excluded owing to missing data caused by poor specimen quality (poor specimen storage, no urine available for collection, or difficulty in collection related to early death or emergency surgery). Therefore, 118 patients were included in the final analysis.

**Fig. 1.**
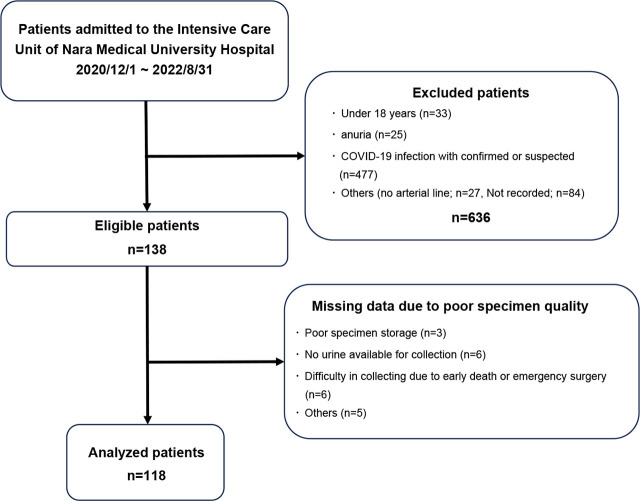
Patient flow chart of the study.

During the 28-day observation period, 101 patients survived, whereas 17 died (Figure 1). Compared with survivors, non-survivors had significantly lower body weight, a lower proportion of external causes of disease, such as trauma (65 of 101 survivors [64.4%] vs. 5 of 17 non-survivors [29.4%]), along with lower albumin levels, and shorter ICU admission times (Table 1). Clinically, non-survivors had significantly higher APACHE II scores, SOFA scores, and arterial lactate concentrations. Additionally, urinary L-FABP concentrations were significantly higher in non-survivors than in survivors at T0 (median 1006.9 vs 24.23 ng/ml, p < 0.001) and T3 (2921.5 vs. 20.69 ng/ml, p < 0.001).

**Table 1.** Baseline clinical characteristics of the study population

**Parameters**	**Survival group (n = 101) median [IQR]**	**Death group (n = 17) median [IQR]**	**p**
Physical data			
Age (years)	65[48–80]	79[75–83]	<0.01
Male, n (%)	61(60.4)	11(64.7)	0.94
Height (cm)	161[154.3–168]	156.5[151–161]	0.09
Body weight(kg)	60.1[50–68.5]	52.6[40–57.8]	0.02

Laboratory data			
WBC (/μL)	11200[8200–16300]	8900[7500–12000]	0.35
Hb (g/dL)	12.2[10.6–13.8]	12[9.9–12.8]	0.30
CRP (mg/dL)	0.14[0.03–1.55]	1.09[0.18–5.33]	0.07
ALB (g/dL)	3.8[3.3–4.2]	3.2[2.8–3.6]	<0.01
Cre (mg/dL)	0.95[0.73–1.45]	1.3[0.89–1.45]	0.17
Lac (mg/dL)	2.1[1.2–3.5]	5.4[2.2–12.9]	<0.01
L-FABP (at admission) (ng/mL)	24.23[5.95–191.04]	1006.9[134.41–7381]	<0.01
L-FABP (3 hours later) (ng/mL)	20.69[7.38–153.96]	2921.5[519.02–6104.5]	<0.01

Scoring data			
APACHE II score	19[14–27]	34[28–40]	<0.01
SOFA score	7[3–10]	11[9–13]	<0.01
Others			
External causes, n (%)	65(64.4)	5(29.4)	0.01
Time to ICU admission (min)	113[86–203]	93[82–107]	0.03

WBC: White blood cell count; Hb: Hemoglobin; CRP: C-reactive protein; ALB: Albumin; Cre: Creatinine; Lac: Lactate; L-FABP: Liver-type fatty acid-binding protein; APACHE II: Acute Physiology and Chronic Health Evaluation II; SOFA: Sequential Organ Failure Assessment; ICU: Intensive Care Unit

**Fig. 2.**
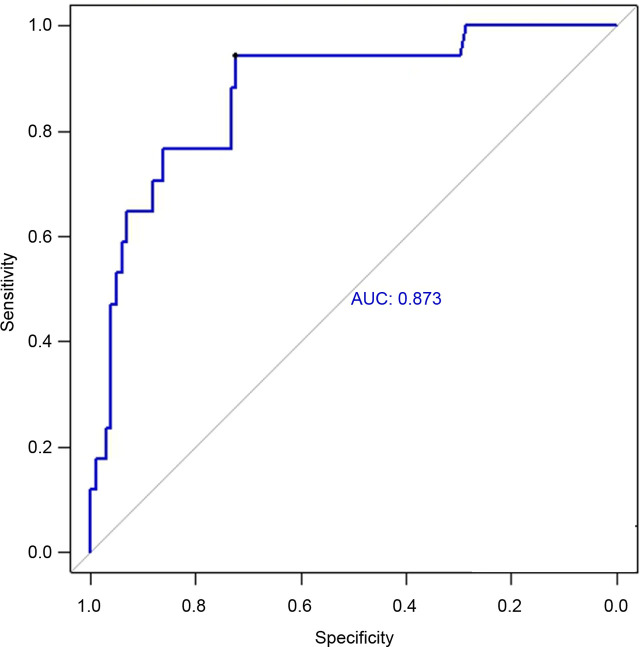
ROC curve for mortality within 28-day prediction by urinary L-FABP at 3 hours after ICU admission. AUC: area under the curve; ICU: intensive care unit; L-FABP: liver-type fatty acid-binding protein; ROC: receiver operating characteristic.

The ROC curve analysis for 28-day mortality (Figure 2) showed that urinary L-FABP at T3 had the highest AUC value (0.8733) compared to L-FABP at T0 (0.7851), APACHE II (0.8008), SOFA (0.7530), and the arterial lactate level (0.7338) (Figure S1). The optimal cutoff value of L-FABP (T3) determined using the Youden index was 90.815.

**Fig. 3.**
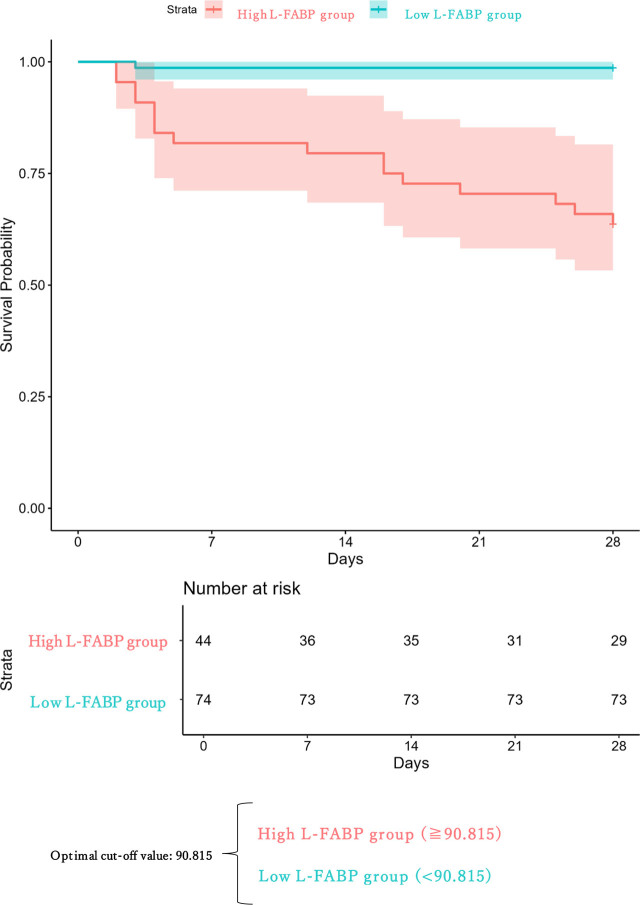
Kaplan-Meier analysis about Survival for 28 days by urinary L-FABP at 3 hours after ICU admission. ICU: intensive care unit; L-FABP: liver-type fatty acid-binding protein.

Based on the cutoff value (T3), the patients were divided into high (H) and low (L) L-FABP groups. The H group consisted of 44 patients (29 survivors and 15 non-survivors), whereas the L group included 74 patients (73 survivors and 1 non-survivor). A Kaplan–Meier survival analysis showed a significant difference in 28-day mortality between the groups (log-rank p < 0.01) (Figure 3).

The crude HR for mortality within 28 days after ICU admission in the High L-FABP group was significantly higher than in the Low L-FABP group (HR: 32.35, 95% CI: 4.29–244.0, p < 0.001). After IPTW, baseline covariates were generally well balanced, with SMDs mostly below 0.1 (Table S1). Although some covariates showed slightly higher SMDs after weighting, the overall balance across clinically relevant variables was substantially improved compared with that in the unweighted sample. After adjusting for confounders using IPTW based on propensity scores for age, gender, weight, albumin level, ICU admission time, lactate level, APACHE II, and SOFA scores, the adjusted HR remained significant (HR: 8.60, 95% CI: 1.02–72.64, P = 0.047) (Table 2).

**Table 2.** Hazard ratio for 28-day survival by urinary L-FABP at 3 hours after ICU admission

**Hazard Ratio for 28-Day Survival**	Low L-FABP* (n=74)	**High L-FABP† (n=44)**	**p-value**
Crude model, HR [95% CI]	1 (reference)	32.35 [4.29–244]	<0.01
IPTW adjusted, HR [95% CI]	1 (reference)	8.60 [1.02–72.64]	0.047

* L-FABP (T3) < 90.815,

† L-FABP (T3) ≥ 90.815;

L-FABP: Urinary Liver-type fatty acid-binding protein; ICU: Intensive Care Unit; HR: Hazard Ratio; CI: Confidence Interval; IPTW: Inverse Probability of Treatment Weighting

In the secondary analysis, the L-FABP ratio (T3/T0) was evaluated using logistic regression to predict 28-day mortality. The Cox proportional hazard ratio was 1.52 (CI: 1.08–2.25, p = 0.0213), and the ROC-AUC (T3/T0) was 0.623 (Figure S2).

## Discussion

This study demonstrated that the urinary L-FABP concentration, particularly when measured at T3, serves as an important and independent predictor of 28-day in-hospital mortality in critically ill patients. Among all evaluated indicators, L-FABP (T3) showed the highest area under the ROC curve (AUC = 0.873) in a descriptive comparison, compared with APACHE II (AUC = 0.801), SOFA (AUC = 0.753), and the arterial lactate level (AUC = 0.734). Furthermore, an elevated L-FABP level at T3 was significantly associated with increased mortality in the IPTW-adjusted Cox proportional hazards model (HR = 8.60, 95% CI: 1.02–72.64, p = 0.047), suggesting that this marker offers potential prognostic value even after adjusting for confounders. However, this estimate should be interpreted with caution, as the number of mortality events was limited (17 deaths), resulting in a wide confidence interval and lower precision of the hazard ratio. The observed magnitude of effect may therefore be statistically unstable, and the true effect size could be substantially smaller or larger.

In contrast, L-FABP levels measured at T0 had a lower predictive performance (AUC = 0.785), indicating that initial levels may not fully reflect the severity of illness or impending physiological deterioration. Moreover, the change in L-FABP levels between T0 and T3, represented by the T3/T0 ratio, demonstrated only modest predictive value (AUC = 0.623; HR = 1.008, 95% CI: 1.006–1.011, p < 0.05), suggesting that the absolute concentration at T3 provides more reliable prognostic information. One possible explanation is that the T3 value reflects not only the baseline disease burden but also the patient’s physiological response to early ICU interventions such as fluid resuscitation, mechanical ventilation, or vasopressor support.

Semiquantitative changes in urinary L-FABP levels within the first 6 hours of ICU admission are related to outcomes [8]; however, the optimal timing and method for evaluating these dynamics remain unclear. Our findings suggest that a single measurement at T3 may offer clinically useful and practical prognostic information, potentially offering an efficient alternative to serial monitoring. From a practical perspective, commercially available semi-quantitative urinary L-FABP point-of-care assays, such as RENAPRO^®^ L-FABP Test POC (CMIC Pharmaceutical Services Co., Ltd., Tokyo, Japan), classify results using predefined thresholds, including ≥100 ng/mL. The cutoff value identified in our study (90.815 ng/mL) falls close to the upper threshold of these assays, suggesting potential clinical compatibility [8]. Importantly, the use of semi-quantitative kits allows rapid assessment within approximately 15 minutes, which may facilitate early risk stratification in emergency and critical care settings.

Biologically, L-FABP is primarily synthesized in hepatocytes, released into the bloodstream, and subsequently excreted in the urine under conditions of oxidative stress and tissue hypoxia. With a short plasma half-life of approximately 11 minutes [9], L-FABP reflects dynamic physiological changes and has been recognized as a sensitive marker of acute kidney injury [10,11,12,13], intestinal ischemia [13], and systemic inflammatory states [14]. Unlike other markers that require longer integration periods, L-FABP offers near-real-time insight into cellular injury, making it especially suitable for critical care settings, where patient conditions can deteriorate rapidly.

Previous studies have demonstrated that elevated urinary L-FABP levels are associated with both short- and long-term mortality in ICU populations [8, 15, 16]. However, most of these studies focused on patients in the medical ICU or on those with chronic comorbidities. In contrast, our study primarily included patients admitted via the emergency department for acute external causes such as trauma. This patient population likely had fewer underlying chronic conditions, thereby reducing confounding and highlighting the role of L-FABP as a marker of acute systemic physiological stress rather than chronic disease burden. Given the heterogeneous and rapidly evolving nature of emergency ICU admissions, biomarkers such as L-FABP, capable of capturing real-time organ dysfunction, may offer particular value in this clinical context.

These findings are particularly relevant in open ICU settings, where intensivists may not be consistently available and critically ill patients are often managed by physicians from diverse specialties such as emergency medicine, internal medicine, surgery, and anesthesiology. In these environments, frequently constrained by time and staffing, simple, rapid, and bedside-accessible biomarkers, such as urinary L-FABP, can support timely risk assessment and facilitate clinical decision-making.

Previous studies have shown that 24/7 in-house ICU intensivist models, as seen in high-acuity, high-volume centers where intensive care specialists provide care on a full-time basis, are associated with better patient outcomes compared to ICUs without dedicated intensivist staffing [17]. These findings suggest that intensivist-led care improves outcomes, likely through the consistent application of evidence-based practices. However, many healthcare institutions worldwide operate under open ICU systems, where access to intensivists is limited. In such contexts, objective and easily interpretable biomarkers such as L-FABP may help bridge the gap by providing real-time assessments of illness severity. Moreover, unlike conventional scoring systems such as APACHE II, which require the integration of multiple clinical variables and time-consuming calculations, urinary L-FABP can be measured from a single-spot urine sample. This noninvasive, rapid, and widely deployable test offers potential utility not only in the ICU but also in emergency departments, general wards, and prehospital settings. By offering a real-time snapshot of a patient’s physiological burden, L-FABP may serve as a triage tool to determine ICU admission or as an objective criterion for patient transfer to higher-level critical care facilities. Its simplicity and portability make it especially attractive for use in resource-limited settings and during early phases of care where time-sensitive decisions are crucial. Future prospective multicenter studies involving heterogeneous ICU populations, as well as interventional trials evaluating L-FABP-guided triage protocols, are warranted to validate and expand upon these findings.

This study has some limitations. First, its retrospective single-center design limits the generalizability of the findings. Our ICU primarily admits patients directly from the emergency department, a population with a relatively high proportion of trauma and other acute external causes. Therefore, patient characteristics, admission protocols, staffing models, and ICU organization may differ from those of medical or elective ICUs, potentially influencing urinary L-FABP levels and their prognostic performance. For example, the higher prevalence of trauma-associated acute kidney injury may have influenced baseline urinary L-FABP levels. Second, the sample size was relatively small (n = 118), partly because of the exclusion of patients with confirmed or suspected COVID-19 during the study period. To ensure safety and prevent infection risk during specimen collection and handling, patients with fever or respiratory symptoms were excluded unless COVID-19 was definitively ruled out. This may have introduced selection bias and reduced the diversity of the study population. Third, most enrolled patients were admitted from the emergency department for external causes such as trauma, burns, or poisoning. Consequently, patients with endogenous causes, such as sepsis or chronic disease exacerbations, were underrepresented. This imbalance may limit the applicability of our findings to other ICU populations, particularly those dominated by medical rather than surgical or trauma cases. Finally, although we used IPTW to adjust for measured confounders, the observational design leaves room for residual confounding from unmeasured variables. Further multicenter, prospective studies involving larger and more heterogeneous populations are warranted to confirm urinary L-FABP’s prognostic value.

## Conclusion

This study demonstrated that the urinary L-FABP concentration measured 3 hours after ICU admission is an important predictor of 28-day mortality and showed a higher prognostic performance than conventional scoring systems such as APACHE II and SOFA in terms of AUC values. Given its simplicity, non-invasiveness, and rapid availability, L-FABP may serve as a practical tool for early risk stratification in critically ill patients. Further prospective studies are warranted to validate its broader clinical applicability across different ICU settings.
